# Effects of cushion box and closed let‐down ladder usage on impact damage to corn kernel during handling

**DOI:** 10.1002/fsn3.3137

**Published:** 2022-11-14

**Authors:** Reza Shahbazi, Feizollah Shahbazi

**Affiliations:** ^1^ Department of Biosystems Engineering Lorestan University Khoram Abad Iran

**Keywords:** corn, drop height, drop method, handling, mechanical damage

## Abstract

The main purpose of this study was to evaluate the effects of the cushion box and closed let‐down ladder usage in minimizing mechanical damage to corn kernels during free fall. Kernels from a single lot of cultivar KSC 705 were evaluated for percentage of breakage using three drop methods (free fall, with cushion box, and with closed let‐down ladder) at five different moisture contents (10%, 15%, 20%, 25%, and 30%), and three drop heights (5, 10, and 15 m). The results showed that the drop methods had a significant effect on the breakage sensibility of kernels. Sample kernels dropped without a ladder (free fall) had a significantly higher average percentage breakage of 13.80%. In the use of the cushion box, the average kernel breakage was calculated to be 11.41%, which was decreased by about 17% more than the free fall. Sample kernels dropped with the closed let‐down ladder had a lower average breakage of 7.26%, which showed that the closed let‐down ladder significantly helped to reduce mechanical damage to corn kernels by about 47% comparing free fall and by about 37% than the use of the cushion box. The amounts of kernel damage increased significantly with increasing drop height and decreasing moisture content, but the use of the cushion box and closed let‐down ladder systems somewhat reduced the adverse effect of the above factors. To minimize mechanical damage to kernels as they fall into the bin, a grain let‐down ladder should be installed in the bin so that it can receive kernels from the filling spout with minimum damage. Empirical models were developed for the dependency of damage to corn kernels due to the impact caused by free fall on the drop height and moisture content at different drop methods.

## Graphical abstract

The article presents essential information for developing knowledge in the quantitative assessment of damage to corn seeds related to mechanical impact injuries, minimization of which may contribute to the reduction of losses during harvesting.

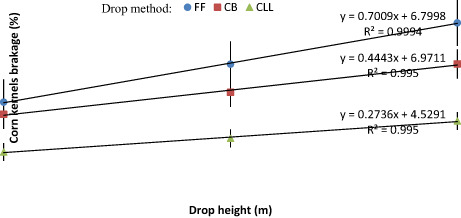

## 1 INTRODUCTION

One of the most important factors that affect the quality of agricultural products is mechanical damage to the grains (seeds). Mechanical injuries can be caused by any type of physical and mechanical action, but injuries caused by the impact of moving parts of machines during harvesting and postharvest processing are the most serious (Shahbazi, 2011). The grains of agricultural products are constantly subjected to impact forces from machines from the moment they are harvested to the time they are transferred into storage (Shahbazi et al., 2011a, 2011b; Shahbazi, Dolwlatshah, & Valizadeh, 2012; Shahbazi, Valizadeh, & Dolatshaie, 2012). Improper design and performance of machines in each of these stages can cause mechanical damage. Damage to grains caused by the impact during these processes is a major problem in the grain industry. Symptoms of mechanical damage to grains may be in several different forms (Chen et al., 2021; Shahbazi, 2021). External damage to the grains is easily visible, such as breakage and cracking of the grain (seed), internal damage (such as the formation of internal cracks), microscopic fissures, and injuries to the embryo of the grain. All these damages reduce the value of the product, reduce the shelf life of the product, create health problems, increase production and processing costs, reduce the efficiency of nutrient extraction from the grains, and reduce the percentage germination and seed vigor.

In addition, depending on the quality of the harvest and the postharvest process, the broken (fine) grains can contain different types of spoilage that may adversely affect grain management in silo bins (Liu et al., 2012; Meyers & Hollinger, 2004). Mechanical damage to the grains reduces the shelf life of the grains by producing carbon dioxide gas and reducing the dry weight of the grains. Fractures, cracks, and scratches on the grain cause air and moisture to penetrate it and cause rapid hydration of the living tissues of the grain, which reduces the ability to store the grain (DaSilva et al., 2018). Another problem of mechanical damage to grains is the uneven distribution of broken particles inside the tank or silo. Uneven distribution of broken particles occurs when the grain is discharged from the top into the silo. Since broken grains do not have a uniform shape, or smooth and uniform surface, compared with healthy grains, they do not spread evenly inside the tank. The uneven distribution of these fine particles and their concentration in one part of the silo bin (mostly in the central part of the bin) causes the nonuniform distribution of airflow paths during aeration or drying of grains and also does not distribute moisture and heat uniformly inside the bin, which causes nonisothermal and humidity points inside the bin and increases the risk of fungal and insect growth (Deng et al., 2021; Fan et al., 2017; Narendran et al., 2019).

Free fall is one of the most important stages in grains and fruits processing and handling, in which products are damaged by impact. Grains are exposed to impact when free falling or spouting onto a hard surface, such as when unloading from a combine harvester into a cart or filling or filling a storage bin (Chen et al., 2020). Depending on the type of operation, grains may fall freely from a few meters on the farm when unloaded by combine harvester and truck transport bin, to a height of more than 15 m when unloaded and loaded in silo bins and grain transport and export terminals (Chen et al., 2020; Shahbazi, 2021). During these stages, the grains fall from different heights and impact various surfaces (concrete, metal, and grain on grain), resulting in potential damage. During conditioning, grains may be stored in bins for short periods until they are ready to be processed. Loading grains into bins may cause mechanical damage as the grains are subjected to a free‐fall drop. The damage to the grains during free fall depends on various factors, such as drop height, grain conditions (grain size, volume, mass, temperature, and moisture content), and contact surface material (Delfan et al., 2022). Using vertical pipes, in silos and other grain handling or processing systems allows the grains to move at high speeds resulting in a high‐intensity impact on the grains when discharged, which may result in mechanical damage to the grain.

To reduce the mechanical damage to the grains caused by free fall, the condition of the grains and their falling conditions should be adjusted so that the severity of the impact is reduced. Given that the conditions of the grains are somewhat uncontrollable during the movement and free fall, the only factor that can be managed and controlled is falling conditions. As much as possible, the drop height and speed of the falling kernels must be reduced to prevent the grains from being hit on hard and winning surfaces. To reduce the drop height and speed of falling of the grains when leaving the conveyors or in unloading or loading the grains storage bins, stepping falling systems (ladder) should be used (Shah et al., 2001). Two kinds of such systems, known as cushion box and closed let‐down ladder (Gregg & Billups, 2016), are commonly used. These types of equipment can be installed in the path of grain transfer pipes or in the center of the grain storage bins to unload or load bins while preserving the desired grains in a safe state without damage (Gregg & Billups, 2016; Shahbazi & Shahbazi, 2022). Also, the most severe impacts between grain and hard surfaces during conditioning can be eliminated or reduced by minimizing drops and/or padding points of impact.

Impact damage to seeds has been the subject of much research due to reduced crop quality during harvesting, handling, and processing. Various test methods have been proposed to predict the crushing strength of corn kernels, including the compression method (Su et al., 2019a, 2019b, 2020), the drop method (Li et al., 2009), the pendulum method (Srivastava et al., 1976), and breakage sensibility (Bilanski, 1966). Many indexes and methods have been developed to determine corn kernel susceptibility to breakage. The breakage susceptibility index is the most widely used and is defined as the likelihood of kernel fragmentation occurring when kernels are subjected to impact forces during handling and transport. Methods for determining breakage susceptibility can be classified into four categories based on the different external forces applied to grain and contact position. These include grain impacts against nongrain surfaces, grain‐on‐grain impacts, rubbing impacts, and centrifugal impacts. The instruments used to determine breakage susceptibility are typically the Wisconsin breakage tester and the Stein breakage tester (Shahbazi, 2021).

Various drop tests were performed with corn, chickpea, and soybean (Bartkowiak et al., 2019; Delfan et al., 2022; Shah et al., 2001). In a study on the improvement of grain quality by Bartkowiak et al. (2019), they reported that corn grains with a moisture content of about 18% dropped using a cascade chute from 6‐m height showed four and five times less damage compared with the free fall. They also reported that a 6‐m drop height cascade chute considerably decreased the velocity of the filling grains when loading the silo. Delfan et al. (2022) evaluated the percentage breakage of chickpea seeds due to the impact caused by free fall as affected by impact surface (concrete, metal, plywood, and seed‐on‐seed), drop height (3, 6, 9, and 12 m), and seed moisture content (10%, 15%, 20%, and 25% w.b.) and reported that chickpea seeds dropped onto concrete and metal had significantly the highest means of percentage breakage of 13.89% and 12.94%, respectively, in comparison with 10.64% and 8.34% on plywood and on seed to seed, respectively. Increased drop height from 3 to 12 m caused a significant increase in the mean values of damage to seeds from 7.20% to 15.57%. Increased moisture levels caused a decreasing trend by a factor of 2 in the damage to seeds due to free fall. Shah et al. (2001) reported that bins filled with soybeans using a bean ladder had a lower percentage of damaged or cracked beans and exhibited a higher quality, including germination levels, than seeds in the bin filled without a ladder.

Bergen et al. (1993), in a study on damage to ‘Trapper’ peas and ‘Laird’ lentils in free fall, observed that seeds dropped from a greater height caused more seed damage on all three selected surfaces, namely, steel, plywood, and concrete. Seeds with lower moisture content reportedly incurred more damage. The result of the study by Shahbazi and Shahbazi (2019) showed that corn kernels with the moisture of less than 18% are very susceptible to mechanical damage under impact loading. Gu et al. (2019) reported that the optimum moisture content for harvesting and threshing two types of corn cultivars of dent and flint, to produce seeds with high germination percentages, were about 15% and 18%, respectively.

Corn is one of the most important products in providing the protein needed by society. Corn kernels have a high susceptibility to mechanical damage because of their large size and mass (Guo, 2015; Li et al., 2020). In addition, corn kernels are very fragile due to the lack of gluten, which is a natural internal binder in the grains (Bartkowiak et al., 2019). Serious mechanical damage during mechanical harvesting and processing has become the primary factor that affects the quality of corn kernels (Su et al., 2021).

There is little published information on the effects of the free fall, cushion box, and closed let‐down ladder on the corn kernel damage (breakage susceptibility) ratio related to different moisture contents and drop heights. Therefore, the objectives of this study were (1) to qualify and quantify the amount of mechanical damage to corn kernels during handling caused by free fall; (2) to develop of cushion box and closed let‐down ladder for decreasing the grains damage in the course of harvesting and postharvest processing; (3) to evaluate the effects of the cushion box and closed let‐down ladder usage in minimizing mechanical damage to corn kernels during free fall, related to moisture content and drop height; and (4) to determine the velocities of kernels dropping from various heights and recommend safer heights for the design of handling equipment.

## 2 MATERIALS AND METHODS

In this study, mechanical damage to corn kernels due to free fall was investigated. The corn kernels used in this study were of a KSC 705 hybrid and were manually harvested from a field located in Khorramabad at the commercial ripening stage and then transferred to the laboratory and carefully selected for homogenous size, shape, and lack of damage (defects). During harvesting, corn kernels had an average moisture content of 31.28%. Samples were stored during the experiment at 5°C and 85%–90% humidity, until the time of these experiments. Physical properties of kernel samples, including the length (*L*), width (*W*), and thickness (*T*), were measured using a digital micrometer, with an accuracy of 0.01 mm. Then, their geometric mean diameter (*Dg*) and sphericity (*φ*) were computed from the data of length, width, and thickness parameters by using the following relationships, respectively (Mohsenin, 1986):

(1) $$D_{g}={\left(\mathrm{LWT}\right)}^{1/3}$$

(2) $$\phi =\frac{{\left(\mathrm{LWT}\right)}^{1/3}}{L}$$

The mass of kernels was measured with a digital scale with an accuracy of 0.01 g. A universal testing machine (Santam ST‐1, Santam Company Tehran, Iran) equipped with a 1000 N load cell was used to determine the mechanical properties (rupture force and displacement (deformation)) at their rupture point following the test procedure described in the ASABE standard S368.3 (ASABE, 2008). The individual kernels were placed between parallel plates of the machine and compressed at a constant loading rate of 1.25 mm/min until rupture. The rupture force and displacement (deformation) at the rupture point for kernels in each experiment were obtained from the force–deformation curve plotted by the machine software (Santam company, Tehran, Iran). The physical and mechanical properties of the grains were measured at the standard moisture content of 15% (Kim et al., 2002; Paulsen et al., 2019). The moisture contents of corn kernels were determined according to ASABE standard S352.2 (ASABE, 2006) and calculated on a wet mass basis. The initial moisture content of the kernels was approximately 10%. Higher moisture levels of 15%, 20%, 25%, and 30% were obtained by spraying precalculated distilled water on kernels spread on plastic sheets. The moisture in kernels was allowed to equilibrate at a temperature of 4°C for at least 10 days.

**FIGURE 1:**
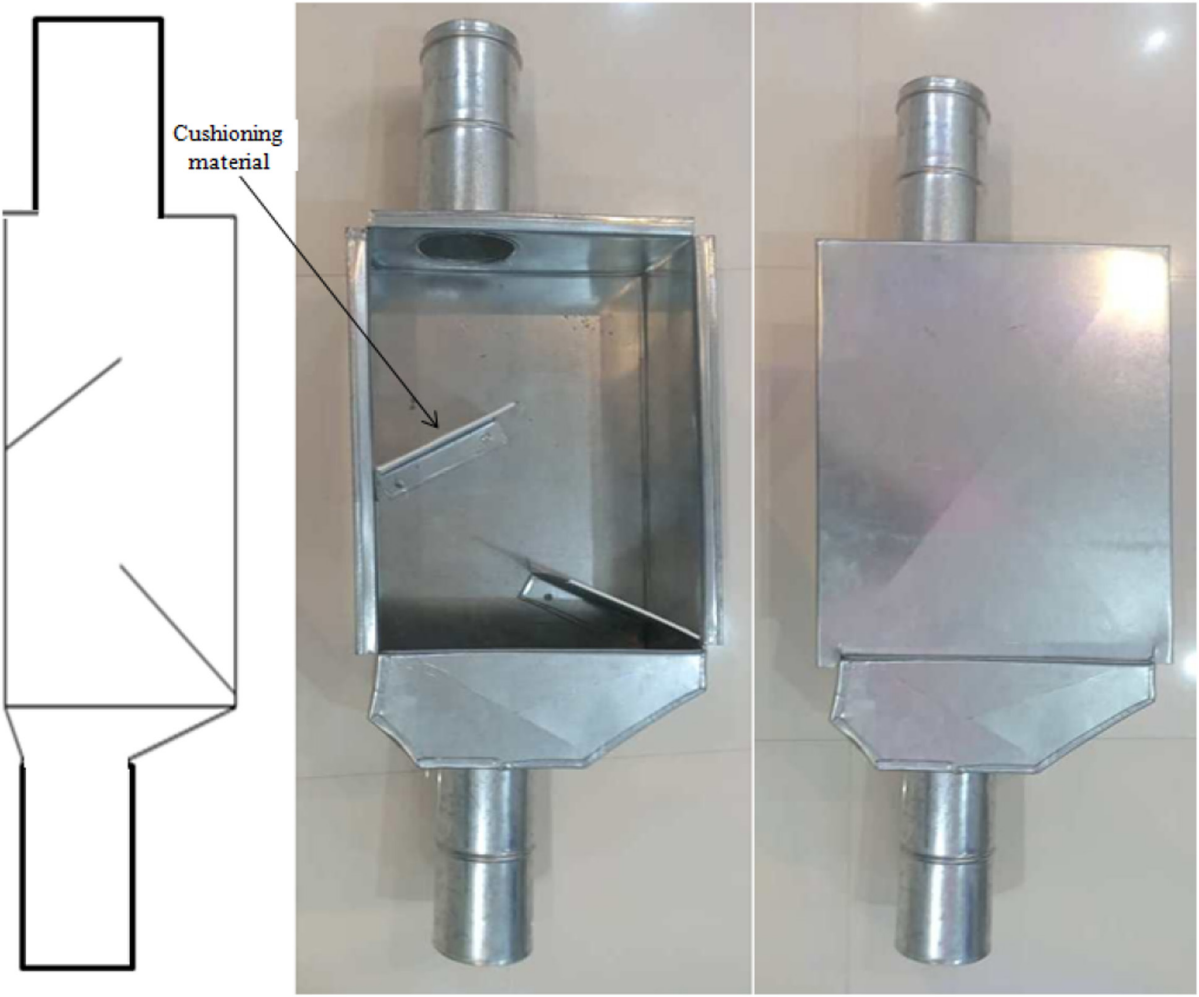
Schematic and the sample of the cushion box used in research.

**FIGURE 2:**
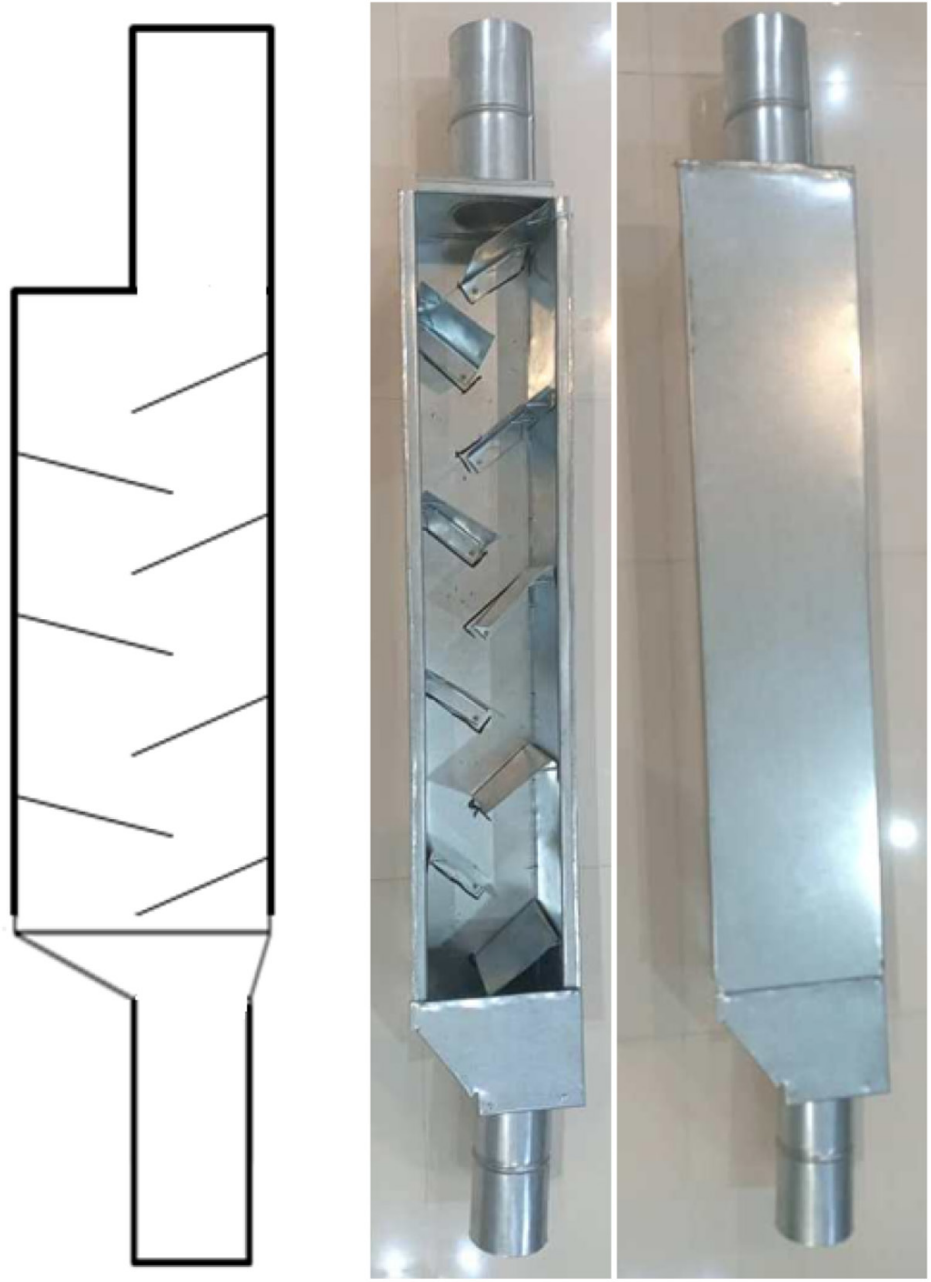
Schematic and the sample of the closed let‐down ladder.

Laboratory tests were used to simulate free fall and evaluate the effect of the cushion box and closed let‐down ladder usage in minimizing mechanical damage to corn kernels. Two dropping systems (cushion box and closed let‐down ladder) were designed and developed. Figure 1 shows the schematic and the sample of the cushion box used in this research. This system consists of a box 100 cm in length and 20 cm in width (twice the width of the transfer pipe diameter (10 cm)). Inside the box, there are two sloped blades 22 cm in length, installed at 45° in the opposite directions of kernels flow and cushioned with rubber with a thickness of 4 mm. Isoprene Rubber (IR) (Bita Rubber Company, Iran), with a density of 0.92 g/cm^3^, elongation at break of 400%–800%, a tensile strength of 17 MPa, and wear resistance index of 120 mg/mm^3^, reported by the manufacturer, was chosen as the cushioning material. Based on the grain transfer pipe length, the cushion box can be installed at different distances along the pipe to minimize mechanical injury caused by impact. The box was made of galvanized steel with a thickness of 7 mm. Figure 2 shows the closed let‐down ladder which has sloped rubber‐padded baffle plates. This type of system lets kernels fall gently to the bin without causing a mechanical impact on the kernels. The structure of this system consists of a cubic box that is 100 cm long and 15 cm in width (1.5 times the width of the transfer pipe diameter (10 cm)). The ladder was made of a galvanized steel sheet with a thickness of 7 mm.

**FIGURE 3:**
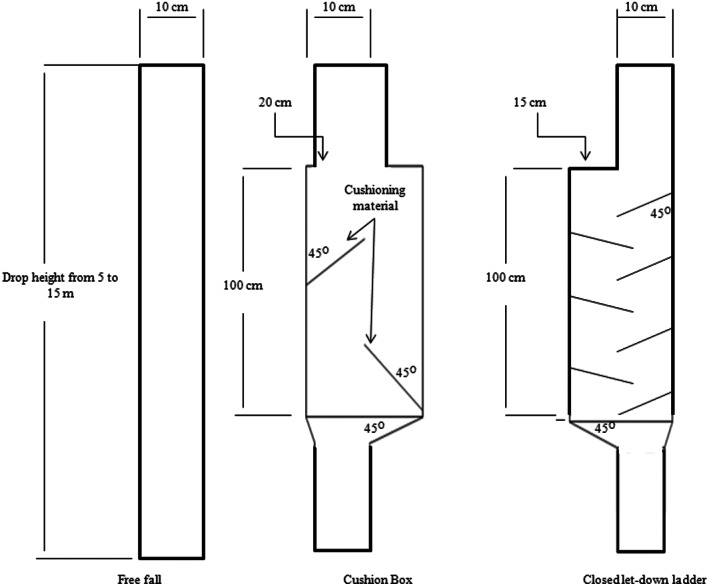
Schematic of three systems used for drop tests.

Kernel samples with moisture contents of 10%, 15%, 20%, 25%, and 30%, which are typical moisture contents at harvest and postharvest operations of corn, were dropped using three different methods: (1) with a cushion box, (2) with a closed let‐down ladder, and (3) with free fall using PVC pipe (with a diameter of 10 cm) without a device (Figure 3). The higher levels of moisture contents of 25% and 30%, which are close to the average value of moisture content of corn kernels during harvesting, were chosen to investigate the effects of moisture on the damage to the kernels during harvesting and handling.

Three drop heights of 5, 10, and 15 m were selected. These drop heights are typically indicative of a situation on the farm, at a grain cleaning plant, or in a grain elevator. For obtaining different drop heights, PVC pipes of 10 cm diameter were set up. A hopper with a 40‐mm‐diameter opening was attached to the top of the pipe. The dropping rate of kernel samples was regulated using a gate attached at the bottom of the hopper. The flow rate of kernel samples was regulated at 0.25 kg s^−1^. For drop tests, 5 kg of pre‐sieved kernels samples (whole kernels without damage) were placed in the hopper and then dropped into the desired system. To prevent the scattering of the grains at the end of the systems, a wooden chamber was placed at floor level. The concrete floor of the chamber was installed at an inclination of 45° to simulate a drop in an empty bottom bin. After drop tests, the samples were then collected from the chamber and transferred to plastic bags for damage analysis.

### 2.1 Damage assessment

According to the method of assessing the quality of grains and cereals (USDA, 2013) on the purchase and sale of agricultural products under public intervention, the quality of corn is assessed using the fraction method and sieve with a round hole of 4.76 mm (12/64 inch). These regulations define damaged grains as unusable grains for animal feed due to rot, mold, bacteria, or other reasons. For damage assessment, the samples were screened using a 4.76 mm (12/64 inch) round hole sieve (USDA, 2013) for the separation of broken kernels. The percentage breakage (breakage susceptibility) of corn kernels was expressed using the following relationship (USDA, 2013):

(3) $$\mathrm{BS}=\frac{\mathrm{Wi}-\mathrm{Wr}}{\mathrm{Wi}}\times 100$$

where *BS* is the percentage breakage (breakage susceptibility). *Wi* is the initial weight of the test kernels sample. *Wr* is the weight of kernels retained by sieve.

### 2.2 Measurement of corn kernels velocity

The velocities of the kernels were measured by dropping a single corn kernel at a time and by dropping the kernels in mass flow. A video camera was used to record the kernel velocity just before hitting the concrete floor of the chamber. A distance of approximately 1 m was maintained between the bottom end of the desired drop system and the chamber floor to record the velocity of the kernels leaving the desired drop system. A set of horizontal lines were drawn at an interval of 5 cm and placed in the background for ease in observing the distance traveled by seeds. Images of both streams of kernels and the individual kernel were taken to observe the air resistance that affected their speed. Because tracking kernels in mass flow was difficult, the velocities of kernels at the beginning and the end of mass flow were recorded.

### 2.3 Statistical analysis

The factorial experiment was conducted as a randomized design. The main effects were the dropping method (free fall, cushion box, and closed let‐down ladder), moisture content (10%, 15%, 20%, 25%, and 30%), and drop height (5, 10, and 15 m). There were three replications for measurements of the percentage breakage of corn kernels (the dependent variable). Thus, there were 135 (3 × 5 × 3 × 3) observations. The main treatments and their interactions were analyzed using analysis of variance (ANOVA) by using SPSS software (version 19). For graphs and tables, Microsoft Excel was used. The level of significance was shown as *p < .05 and **p < .01 by applying Duncan's multiple range tests.

## 3 RESULTS AND DISCUSSION

In this study, mechanical damage to KSC 705 corn hybrid due to free fall and the effects of the drop method, drop height, and moisture content was investigated. Table 1 describes the physical and mechanical properties of the corn kernels.

**TABLE 1:** Physical and mechanical properties of the studied corn kernels

Measured properties							
Length (mm)	Width (mm)	Thickness (mm)	Geometric mean diameter (mm)	Sphericity	Mass (g)	Rupture force (N)	Deformation (mm)
10.14 (1.05)^a^	7.64 (0.52)	5.17 (0.82)	7.33 (0.33)	13.14 (2.09)	0.29 (0.03)	229.52 (80.52)	0.35 (0.13)

^a^ Standard division.

The results of the study illustrated that the percentage breakage of corn kernels was affected by the drop method, drop height, and moisture content. Table 2 shows the results of the analysis of variance for the percentage breakage of corn kernels in different treatments of drop method, drop height, and moisture content. The drop method, drop height, and moisture content appeared to have significant effects on the percentage breakage of corn kernels (*p* < .01). Still, the impact of the drop method on the percentage breakage of corn kernels was more (*F* = 770.61) followed by moisture content (*F* = 744.43) and drop height (*F* = 394.04) within the range of variables studied (Table 2). Furthermore, the interaction between drop method × drop height, drop method × moisture content, and drop height × moisture content had significant effects (*p* < .01) on the percentage breakage of corn kernels.

**TABLE 2:** Analyses of variance for the percentage breakage of corn kernels as affected by drop method, drop height, and moisture content.

Source	DF	Sum of squares	Mean square	*F*
Drop method (DM)	2	986.541	493.226	770.611**
Drop height (DH)	2	504.409	252.204	394.042**
DM × DH	4	69.470	17.368	27.135**
Moisture content (MC)	4	1905.871	476.468	744.429**
DM × MC	8	164.601	20.575	32.146**
DH × MC	8	29.983	3.748	5.856**
DM × DH × MC	16	20.370	1.273	1.989*
Error	90	57.64	0.640	
Total	135	19,570.18		

** *p* < .01;

* *p* < .05.

Table 3 shows the means of the percentage damage to corn kernels used in drop tests. All three independent variables, namely, drop methods, drop heights, and moisture content, had a significant effect (*p* = .05) on the measured values.

**TABLE 3:** Duncan's multiple range tests compare the means of the percentage breakage of corn kernels in different treatments of drop method, drop height, and moisture content

	Treatment	Percentage breakage of corn kernels
Drop method	Free fall	13.80 a
	Cushion box	11.41 b
	Closed let‐down ladder	7.26 c
Drop height (m)	5	8.53 c
	10	10.70 b
	15	13.26 a
Moisture content (%)	10	16.72 a
	15	13.25 b
	20	10.08 c
	25	7.69 d
	30	6.40 d

*Note*: a–d: Mean values in the columns with the same letter are not significantly different (*p* < .05).

The percentage breakage of corn kernels significantly decreased by using the cushion box and closed let‐down ladder. In addition, the percentage breakage of kernels decreased as the moisture of kernels increased or the drop height decreased within the tested ranges.

From the data in Table 3, it is evident that there were significant differences between the average percentage damage to the kernels using the different drop methods (*p* < .05). In different test conditions, which included different levels of kernels' moisture content and drop height, sample kernels that dropped using a pipe without a system (free fall) had a significantly higher percentage breakage of 13.80%. In the case of using the cushion box, the amount of breakage was 11.41%, which was a decrease of approximately 17% lower than that of free fall. Sample kernels dropped using the closed let‐down ladder had a significantly lower average percentage breakage of 7.26%, showing that the closed let‐down ladder significantly helped to reduce mechanical damage to the kernel by approximately 47% compared to that of free fall and approximately 37% compared to that of the cushion box, which effectively prevented damage to the kernels and reduced the resulting losses.

No published results exist in the literature showing these same results utilizing a cushion box and closed let‐down ladder usage in minimizing mechanical damage (the percentage breakage) to corn kernels during free fall. The effectiveness of cushion box, spout retarders, and retro‐air retarder in reducing damage was studied by Stephens and Foster (1977). It appeared that the flow decelerators were able to reduce handling damage; however, the degree of reduction was small. Shah et al. (2001) reported that bins filled with soybeans using a bean ladder had a lower percentage of damaged or cracked beans and exhibited a higher quality, including germination levels, than seeds in the bin filled without a ladder. In a study on the improvement of grain quality by Bartkowiak et al. (2019), they reported that corn grains with a moisture content of about 18% dropped using a cascade chute from 6‐m height showed four and five times less damage compared with the free fall. They also reported that a 6‐m cascade chute considerably decreased the velocity of the filling grains when loading the silo.

There were differences (*p* < .05) between the percent damage (the percentage breakage) to kernels when using the cushion box compared to that of the closed let‐down ladder (Table 3).

This could be caused by differences in the design and operation of the above systems. In the cushion box, because of the position of its blades, which are in the opposite direction of the kernels' flow, an additional impact may be created, which could result in more damage to the kernels when compared to the closed let‐down ladder. Additionally, this difference may be caused by differences in the corn kernel's acceleration and average mass flow velocities during dropping using the cushion box and the closed letdown ladder. Therefore, further research is needed to investigate this issue.

The percentage breakage of corn kernels increased significantly with increasing drop height (Table 3). Furthermore, there were significant differences between the average damage to kernels at different levels of drop heights (*p* < .05). In different experimental conditions, including different drop methods and different levels of moisture content, the least damage to kernels (8.53%) was caused at a drop height of 5 m. By increasing the drop height from 5 to 10 m, the mean values of the percentage breakage of kernels increased by1.25 times (about 25%) and increased from 8.53% to 10.70%. At a drop height of 15 m, there was higher damage to corn kernels and the percentage breakage of kernels at this height was equal to 13.26%, which increased by about 55% (by 1.55 times) compared to the drop height of 5 m, and by about 23% (by 1.23 times) compared to the drop height of 10 m.

According to the law of conservation of energy *E* = *mgh*, where *E* denotes impact energy (J), *m* denotes kernel or sample mass (kg), *g* denotes acceleration of gravity (9.81 m/s^2^), and *h* denotes drop height (m), it is predictable that with increasing drop height, the amount of applied impact energy to the kernels will be increased, and, as a result, the amount of damage will increase. Also, this is because of the increasing kernel velocity with drop height, which results in a large impact force (Fiscus et al., 1971). Foster and Holman (1973) reported that when the drop height was more than 15 m, the velocity of the grain stream could exceed the single kernel velocity because, when the grain stream was dropped as a whole, the drag forces applied to the individual grains were not all the same. They (1973) suggested limiting the drop height to 12 m (40 feet) to reduce free fall damage. Therefore, it is necessary to reduce the drop height of the grains as much as possible. One of the ways to do this is the use of ladders systems that were mentioned in the previous section. The adverse effect of increasing drop height was similar to what was reported by Bergen et al. (1993) on ‘Laird’ lentils. Perry and Hall (1966) evaluated the mechanical damage to pea beans using drop tests and observed that the damage to pea beans was found to vary proportionately with drop height. Furthermore, similar results were reported by Fiscus et al. (1971) about mechanical damage to corn, soybeans, and beans. Gatongi (1982) reported that mechanical damage during corn grain processing was affected by drop height and moisture content. Asiedu (1986) reported a sharp decrease in the germination percentage of corn seeds with increasing drop height on hard surfaces.

As the moisture content of the kernels increased, mechanical damage to corn kernels including the percentage breakage of kernels decreased (Table 3), and there were differences between average damages to kernels at different levels of moisture contents (*p* < .05). At lower moisture contents, grains are more brittle and adversely affect the impact resistance; thus, they are more prone to physical damage during free fall and other processes, similarly reported by Bergen et al. (1993) for ‘Laird’ lentils, Evans et al. (1990) for soybeans, and Shahbazi et al. (2017) for green and red lentil seeds.

At different dropping methods and different drop heights, kernels with 10% moisture content had a higher average percentage breakage of 16.72%, compared to 13.25%, 10.08%, 7.69%, and 6.40%, at moisture contents of 15%, 20%, 25%, and 30%, respectively. The adverse effect of decreasing moisture content on corn kernels was similar to what was reported by Shahbazi and Shahbazi (2019), Su et al. (2019a, 2019b), and Gu et al. (2019).

**FIGURE 4:**
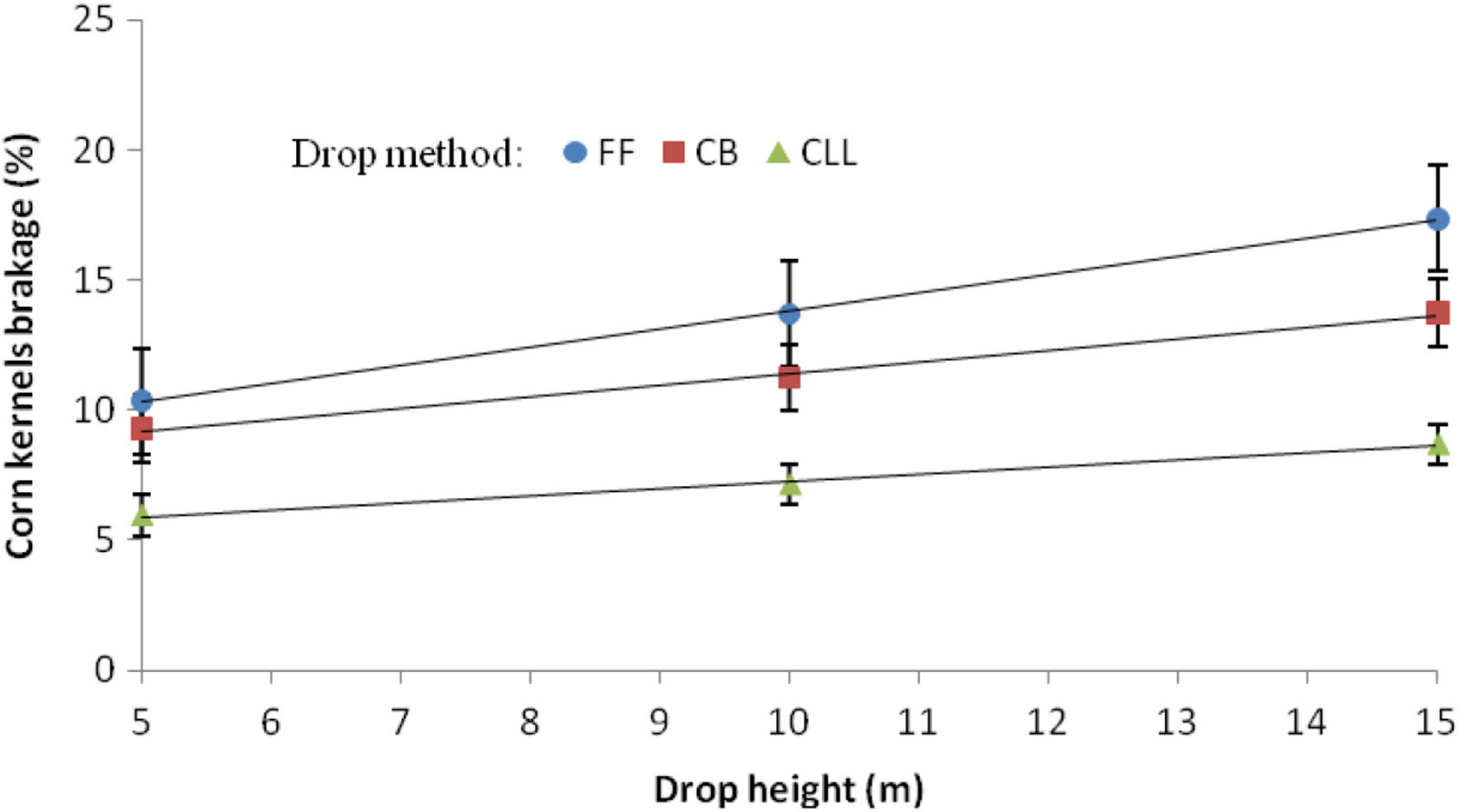
Interaction effect of drop method and drop height on the percentage breakage of corn kernels. CB, cushion box; CLL, closed let‐down ladder; FF, free fall.

Figure 4 shows the interaction effect of the drop method and drop height on the percentage damage to corn kernels. The effect of drop height was highly critical when kernels were dropped using a free fall or cushion box. This difference was higher at the drop height of 15 m compared with the 10 and 5 m heights. The same trend was observed using the closed let‐down ladder, in which the damage was greater at the drop height of 15 m, but the difference in kernels damage at the three drop heights when kernels were dropped using the closed let‐down ladder was significantly lower. In Figure 4, the lowest corn kernel percentage breakage was 5.95%, which was created by the interaction effect of the closed let‐down ladder and the drop height of 5 m. The highest kernel breakage was 17.36%, created in the case of free fall and drop height of 15 m. It is evident that, in all drop heights, the lower damage was caused by the use of the closed let‐down ladder and the highest amount was caused by the free fall. Furthermore, in different drop heights, a medium amount of damage was related to the use of the cushion box. The dependency of breakage susceptibility of corn kernels (*BS*, %) on drop height (*DH*, m) was expressed by the following bets‐fit equations for drop methods of free fall, cushion box, and closed let‐down ladder, respectively:

(4) $$\mathrm{BS}=6.79+0.70\mathrm{DH}\;R^{2}=0.99\;\mathrm{at}\;\text{free fall}$$

(5) $$\mathrm{BS}=6.98+0.45\mathrm{DH}\;R^{2}=0.99\;\mathrm{at}\;\text{cushion}\;\mathrm{box}$$

(6) $$\mathrm{BS}=4.23+0.27\mathrm{DH}\;R^{2}=0.99\;\mathrm{at}\;\text{closed}\;\mathrm{let}‐\text{down ladder}$$

The regression statistics for the models indicated that all the indexes (terms) were significant at the level of 99.99% on the accuracy of the models. As follows from the relations ((4), (5), (6)), the effect of drop height (*DH*) is stronger on the breakage susceptibility of corn kernel (*BS*) at the drop method of free fall (higher indexes at free fall) than cushion box and closed let‐down ladder, showing that the use of the cushion box and closed let‐down ladder systems somewhat reduced the adverse effect of the drop height on the percentage breakage of corn kernels due to free fall.

**FIGURE 5:**
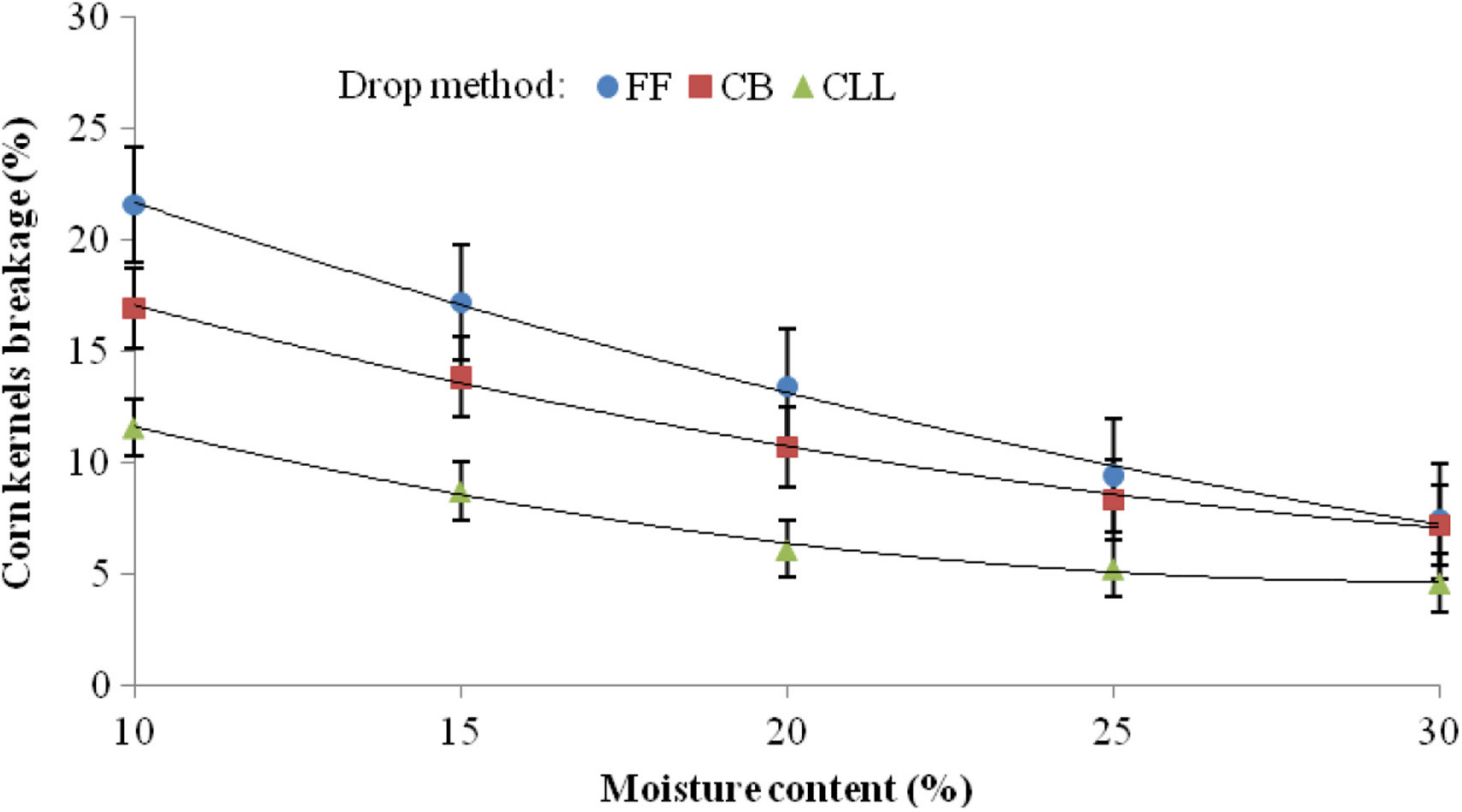
Interaction effects of drop method and moisture content on the percentage breakage of corn kernels. CB, cushion box; CLL, closed let‐down ladder; FF, free fall.

Shown in Figure 5 is the interaction effect of the drop method and moisture content on the percentage breakage of corn kernels. In all the drop methods, as the moisture level decreased, the damage to kernels increased. The difference in kernels damage at the five moisture levels when kernels were dropped using the closed let‐down ladder was significantly lower than the kernels that dropped using free fall. The effect of the moisture level was less critical when seeds were dropped using the closed let‐down ladder. The lower damage was 4.62%, which was created by the interaction of the use of the closed let‐down ladder and moisture content of 30%. The highest damage was 21.67%, which was created in the case of free fall with a moisture content of 10%. In all‐grain moisture levels, the medium damage was related to the use of the cushion box. The dependency of breakage susceptibility of corn kernel (*BS*, %) on moisture content (*MC*, %) was expressed by the following bets‐fit equations for drop methods of free fall, cushion box, and closed let‐down ladder, respectively:

(7) $$\mathrm{BS}=32.88-1.25\mathrm{MC}+0.014{\mathrm{MC}}^{2}\;R^{2}=0.99\;\mathrm{at}\;\text{free fall}$$

(8) $$\mathrm{BS}=26.08-1.03\mathrm{MC}+0.013{\mathrm{MC}}^{2}\;R^{2}=0.99\;\mathrm{at}\;\text{cushion}\;\mathrm{box}$$

(9) $$\mathrm{BS}=20.41-1.02\mathrm{MC}+0.012{\mathrm{MC}}^{2}\;R^{2}=0.99\;\mathrm{at}\;\text{closed}\;\mathrm{let}‐\text{down ladder}$$

The regression statistics for the models indicated that all the indexes (terms) were significant at the level of 99.99% on the accuracy of the models. As follows from the relations ((7), (8), (9)), the effect of moisture content (*MC*) is stronger on the breakage susceptibility of corn kernel (*BS*) at the drop method of free fall (higher indexes at free fall) than cushion box and closed let‐down ladder, showing that the use of the cushion box and closed let‐down ladder systems somewhat reduced the adverse effect of the moisture content on the percentage breakage of corn kernels due to free fall.

**FIGURE 6:**
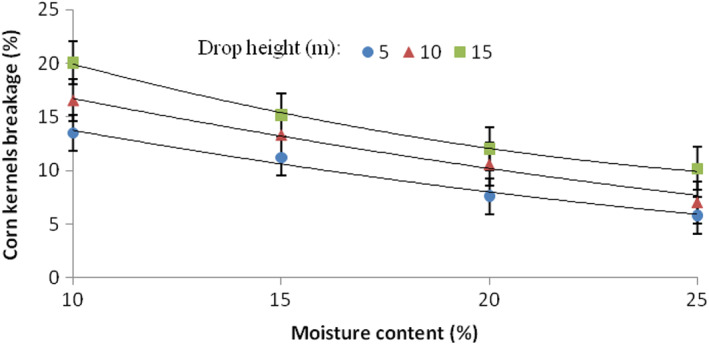
Interaction effects of drop height and moisture content on the percentage breakage of corn kernels.

Figure 6 shows the interaction of drop height and moisture content on the corn kernel breakage. As the moisture level decreased, the damage to kernels increased at a higher rate with the increase in drop height.

The velocities of the seeds were observed by dropping one kernel at a time and by dropping the seeds in a mass flow. Table 4 shows the average velocities (single kernel and mass flow) for corn kernels dropped from various heights using the three different drop methods. In the free‐fall drop method, the average velocities (single seed and mass flow) measured were as expected much higher compared to dropping the kernels using either the cushion box or the closed let‐down ladder. Also, kernels dropped using the closed let‐down ladder had lower velocity values compared to the cushion box at all three drop heights. In addition, in different drop methods, mass flow velocity measurement, velocities were higher compared to dropping the kernels individually. Kernels dropped individually had lower velocity values which may be due to the effect of air resistance encountered in the drop tubes.

**TABLE 4:** Average velocities for kernels dropped from various heights in three drop methods

Drop height (m)	Drop method					
	Free fall		Cushion box		Closed let‐down ladder	
	Velocity (single kernel) (m/s)	Velocity (mass flow) (m/s)	Velocity (single kernel) (m/s)	Velocity (mass flow) (m/s)	Velocity (single kernel) (m/s)	Velocity (mass flow) (m/s)
5	7.25	7.85	4.85	5.32	3.2	3.65
10	9.45	10.12	7.36	8.45	4.65	5.03
15	11.02	13.9	9.89	11.13	6.32	7.69

According to the data in Table 4, at different drop methods, the mass velocity of corn kernels increased significantly with increasing drop height increased the percentage breakage of corn kernels. Grain damage increased as the impact velocity increased which was also the case for soybeans (Evans et al., 1990; Paulsen et al., 1981) and chickpeas (Shahbazi, 2011). In free fall, the average mass flow velocities of kernels when dropped from the heights of 5, 10, and 15 m were 7.85, 10.12, and 13.90 m/s, respectively. At these velocities, the average percentage breakage of corn kernels was 10.35%, 13.71%, and 17.62%, respectively (Figure 4). The average mass flow velocities of seeds when dropped using a closed let‐down ladder from the heights of 5, 10, and 15 m were 3.65, 5.03, and 7.69 m/s, respectively, at these velocities, the means of the percentage breakage of corn kernels were 5.95%, 7.15%, and 8.69%, respectively (Figure 4), showing that the lower mass flow velocity (rate) of kernels during the falling was achieved as a result of the application of the closed let‐down ladder (Table 4), resulting in a slower slide of the kernels and thereby limiting the proportion of kernels damaged in the test comparing free fall. Therefore, the innovative system for storing corn kernels allows for its full use in practice. This improves the quality of corn kernels and allows them to be safely stored in silos.

## 4 CONCLUSIONS

In this study, the effects of three drop methods in corn kernel handling (related to moisture content and drop height) on the percentage breakage of corn kernels were compared. From the results obtained, it was observed that at different levels of moisture contents and drop heights, the use of the cushion box and closed let‐down ladder effectively and significantly minimized mechanical damage to corn kernels during free fall. In addition, there was a significant difference between the effects of the cushion box and the closed let‐down ladder. In the use of cushion box, the damage to the kernels was about 17% less than the pipe without a system. In the use of the closed let‐down ladder, the percentage breakage of corn kernels was reduced by about 47%, in comparison with the pipe without a ladder (free fall), and was about 36% lower compared to the use of the closed let‐down ladder, which effectively prevented damage to the kernels and reduced the resulting losses. In addition, the lower mass flow velocity (rate) of kernels during the falling was achieved as a result of the application of the closed let‐down ladder, resulting in a slower slide of the kernels and thereby limiting the proportion of kernels damaged in the test. Therefore, the innovative system for storing corn kernels allows for its full use in practice. This improves the quality of corn kernels and allows them to be safely stored in silos. Drop height was a critical factor. Increased drop height caused increased damage. The least damage to kernels was caused at drop heights of 5 and 10 m. At the drop height of 15 m, higher damage to corn kernels has been caused and the breakage at this height was equal to 13.26%, which was increased by about 36% compared to the drop height of 5 m, and about 20% compared to the drop height of 10 m. As the moisture level decreased, the damage to kernels increased. Kernels with 10% moisture content had a higher mean percentage breakage of 16.72%, compared to 13.25%, 10.08%, 7.69%, and 6.40% at moisture contents of 15%, 20%, 25%, and 30%, respectively. Therefore, the recommendations of this study for the design of the devices in corn kernel harvest and postharvest operations for preventing mechanical damage caused by free fall regarding the condition of the grains and their falling conditions should be adjusted so that the severity of the impact is reduced. For these purposes, drop height should be minimized. Corn kernels showed to be the most resistant to mechanical injury during free fall when the moisture content ranged from 15% to 20%. In addition, to minimize mechanical damage to kernels as they fall into the bin, a grain let‐down ladder should be installed in the bin so that it can receive kernels from the filling spout with minimum damage.

## FUNDING INFORMATION

This research received no external funding.

## CONFLICTS OF INTEREST

The authors declare no conflict of interest.

## ETHICS STATEMENT

This research does not involve human participants.

## PATIENT CONSENT STATEMENT

This research does not involve patient participants.

## PERMISSION TO REPRODUCE MATERIAL FROM OTHER SOURCES

This research does not involve reproducing material from other sources.

## Data Availability

Research data are not shared.
